# Developing and validating an integrated gross tumor volume (GTV)-TNM stratification system for supplementing unresectable locally advanced non-small cell lung cancer treated with concurrent chemoradiotherapy

**DOI:** 10.1186/s13014-020-01704-2

**Published:** 2020-11-10

**Authors:** Nai-Bin Chen, Qi-Wen Li, Zheng-Fei Zhu, Yi-Ming Wang, Zhangkai J. Cheng, Zhou-Guang Hui, Su-Ping Guo, Hao-Qiang He, Bin Wang, Xiao-Yan Huang, Ji-Bin Li, Jin-Yu Guo, Nan Hu, Xin-Lei Ai, Yin Zhou, Da-Quan Wang, Fang-Jie Liu, Chuan-Miao Xie, Bo Qiu, Hui Liu

**Affiliations:** 1 Department of Radiation Oncology, State Key Laboratory of Oncology in South China, Collaborative Innovation Center for Cancer Medicine, Sun Yat-Sen University Cancer Center, 651 Dongfeng Road East, Guangzhou, Guangdong 510060 People’s Republic of China; 2 Medical Imaging, State Key Laboratory of Oncology in South China, Collaborative Innovation Center for Cancer Medicine, Sun Yat-Sen University Cancer Center, Guangzhou, People’s Republic of China; 3 Clinical Research, State Key Laboratory of Oncology in South China, Collaborative Innovation Center for Cancer Medicine, Sun Yat-Sen University Cancer Center, Guangzhou, People’s Republic of China; 4 Department of Radiation Oncology, Fudan University Shanghai Cancer Center, Shanghai, People’s Republic of China; 5 Department of Radiation Oncology, The First Affiliated Hospital of Jinan University, Guangzhou, People’s Republic of China; 6 Department of Radiation Oncology, National Cancer Center/National Clinical Research Center for Cancer/Cancer Hospital, Chinese Academy of Medical Sciences and Peking Union Medical College, Beijing, People’s Republic of China; 7 VIP Medical Services, National Cancer Center/National Clinical Research Center for Cancer/Cancer Hospital, Chinese Academy of Medical Sciences and Peking Union Medical College, Beijing, People’s Republic of China; 8 Evidance Medical Technologies Inc., Ningbo, People’s Republic of China; 9 Lung Cancer Institute, Sun Yat-Sen University, Guangzhou, People’s Republic of China

**Keywords:** Gross tumor volume, The integrated GTV-TNM stratification system, Locally advanced non-small cell lung cancer, Concurrent chemoradiotherapy

## Abstract

**Purpose:**

The gross tumor volume (GTV) could be an independent prognostic factor for unresectable locally advanced non-small cell lung cancer (LANSCLC). We aimed to develop and validate a novel integrated GTV-TNM stratification system to supplement LANSCLC sub-staging in patients treated with concurrent chemoradiotherapy (CCRT).

**Methods:**

We performed a retrospective review of 340 patients with unresectable LANSCLC receiving definitive CCRT. All included patients were divided into two randomized cohorts. Then the Kaplan–Meier method and Cox regression were calculated to access the prognostic value of the integrated GTV-TNM stratification system, which was further validated by the area under the receiver operating characteristic curve (AUC) score and F1-score.

**Results:**

The optimal outcome-based GTV cut-off values (70 and 180 cm^3^) of the modeling cohort were used to determine each patient’s integrated GTV-TNM stratum in the whole cohort. Our results indicated that a lower integrated GTV-TNM stratum could had better overall survival and progression-free survival (all *P* < 0.001), which was recognized as an independent prognostic factor. Also, its prognostic value was robust in both the modeling and validation cohorts. Furthermore, the prognostic validity of the integrated GTV-TNM stratification system was validated by significantly improved AUC score (0.636 vs. 0.570, *P* = 0.027) and F1-score (0.655 vs. 0.615, *P* < 0.001), compared with TNM stage.

**Conclusions:**

We proposed a novel integrated GTV-TNM stratification system to supplement unresectable LANSCLC sub-staging due to its prognostic value independent of TNM stage and other clinical characteristics, suggesting that it could be considered in individual treatment decision-making process.

## Introduction

Lung cancer remains the most common cancer worldwide with the highest cancer-related mortality [1] and non-small cell lung cancer (NSCLC) accounts for approximately 85% of all these cases. The standard treatment of NSCLC depends primarily on the TNM staging system, which has been regarded as the single most prognostic factor in predicting survival outcomes of patients with lung cancer [2]. For patients with early stages diseases, surgical resection is recommended to be the primary therapy owing to its curative effect. The 5-year overall survival (OS) of these patients with complete resection ranges from 29 to nearly 90% [3, 4]. However, nearly 30% of patients with NSCLC have unresectable, locally advanced, stage III diseases, which nominated definitive concurrent chemoradiotherapy (CCRT) as principal treatment modality.

As is well known, stage III NSCLC represents a heterogeneous population, even within the same TNM stage following radical CCRT, there are large variations in survival outcome, suggesting that further pretreatment assessment beyond TNM stage may optimize treatment strategies and then improve clinical outcome. Several potentially important prognostic factors, specific for lung cancer treated with radiation therapy, have been identified, including performance status, weight loss, age, gender and histology [5]. In addition, tumor volume is a promising prognostic indicator revealed in numerous studies, which has been recognized in the carcinoma of head and neck, esophageal cancer, melanoma, breast cancer and cervical carcinoma treated with radiotherapy [6–10]. Previous radiobiological studies [11–14] have demonstrated that a larger tumor is related to more proliferation, hypoxia, and radio-resistance, which may compromise therapeutic effects. However, prognostic value of tumor volume in locally advanced non-small cell lung cancer (LANSCLC) has not yet been widely investigated.

It is generally considered that the current TNM staging system still has limitations, especially in the era of modern radiation technique. Simultaneous modulated accelerated radiation therapy (SMART) is widely used in LANSCLC and allows for precise dose delivery to the irradiated target, so that dose escalation can be achieved to improved disease control without excessive toxicity. The classical TNM staging depends mainly on operability, which focuses more on tumor size and location, and lacks quantitative volumetric evaluation of overall tumor burden. To date it has been difficult in a way to quantify tumor burden directly and accurately, which may restrict its application in clinical practice. With the advent of computerized planning systems for gross tumor target contouring and treatment planning in radiotherapy, data on gross tumor volume (GTV) can be availably and systematically calculated. Thus, we launched a study to investigate prognostic value of GTV and TNM staging in unresectable LANSCLC treated with definitive CCRT, and then develop and validate an integrated GTV-TNM stratification system for clinical practice.

## Materials and methods

### Data collection

Consecutive patients diagnosed with unresectable LANSCLC who were treated with definitive CCRT at our institution between September 2011 and September 2018 were retrospectively screened. The entry criteria were as follows: (1) pathologically confirmed NSCLC; (2) inoperable stage III disease because of bulky primary disease (T3 or T4), mediastinal lymph node involvement (unresectable N2 or N3), or contraindication for surgical resection; (3) treated with radical radiotherapy (total radiation does ≥ 60 Gy) with concurrent chemotherapy; (4) without a history of prior chest radiotherapy; (5) Eastern Cooperative Oncology Group (ECOG) performance status score 0 to 2; (6) follow-up no less than 6 months since the start of radiotherapy unless death or tumor progression was documented. Those who met the above inclusion criteria in our two ongoing randomized clinical trials were enrolled in this analysis. All included patients were randomly divided, with a ratio of 2:1, into a training group and a validation group. Each patient received physical examination, electrocardiogram, pulmonary function test, laboratory tests, computed tomography (CT) scans of the chest and upper abdomen, brain magnetic resonance imaging (MRI), bone scan and/or positron emission tomography/computed tomography (PET/CT) and pathological biopsy. Tumor Staging was re-classified according to the TNM staging system proposed by the American Joint Committee on Cancer (8th edition) based on clinical work-ups.

### Radical radiotherapy and GTV contouring

Patients were positioned supine and immobilized in a vacuum cradle. Using 4-dimensional computed tomography (4DCT) (Brilliance CT Big Bore, Philips), stimulation CT data sets scanning from the Atlas level to the second lumbar vertebra level with 5 mm thickness slices were obtained in 10 respiratory phases, and a maximum intensity projection (MIP) data set was constructed, which were then exported to the planning system (Monaco planning system, Elekta Medical Systems, Stockholm, Sweden) for target contouring and treatment planning. GTV, clinical target volume (CTV), planning target volume (PTV), and organs at risk (OARs) were delineated. The GTV included the primary tumor and positive regional lymph nodes, which were defined as nodes with a short-axis diameter no less than 1 cm on CT scan, or with high fluorodeoxyglucose uptake on PET/CT scan, or pathologically approval by mediastinoscopy or endobronchial ultrasound-transbronchial needle aspiration (EBUS-TBNA). For patients receiving neoadjuvant chemotherapy, the GTV was defined as the post-chemotherapy volume of initially determined primary tumor and positive regional lymph nodes. GTV-lung and GTV-node was contoured using pulmonary or mediastinal window CT settings, respectively. The CTV was defined as a 0.6 cm margin around GTV-lung, involved lymph node region and 1–2 elective stations. PTV-GTV and PTV-CTV was produced by expanding GTV and CTV with a 0.6 cm margin in all directions, respectively. Lungs, esophagus, spinal cord and heart were delineated as OARs. All contours were reviewed by a senior physician. All patients received SMART, using a 6–8 MV photon beam. A radiation dose of 60–70 Gy (2.0–3.0 Gy per fraction) was delivered to PTV-GTV, and 45–50 Gy (1.8–2.6 Gy per fraction) to PTV-CTV, in 24–33 fractions. At least 95% of PTV received 95% of prescribed dose. Dose constraints on the OARs were as follow: V20 < 35% for lungs; mean lung dose < 1 Gy; maximum dose of esophagus < 66 Gy; maximum dose of spinal cord < 46 Gy; V30 < 30% for heart. Throughout the course of radiotherapy, weekly cone beam computed tomography (CBCT) was acquired before radiotherapy delivery for verification of the position of the tumor and OARs.

### Concurrent chemotherapy

Platinum-based double agents were administered for concurrent chemotherapy in most patients. The regimens included docetaxel/ paclitaxel/ pemetrexed/ etoposide plus platinum, weekly or every 3 weeks. Several patients in clinical trial received triple agents as Nimotuzumab included. In patients with intolerance to double agents, single agent included pemetrexed, taxel or tyrosine kinase inhibitor (TKI) was allowed.

### Follow-up and treatment response assessments

Each patient received a chest and upper abdomen CT scan every 3 months for the first 2 years after completion of CCRT, and subsequently every 6 months until tumor progression or death, while brain MRI was required every 6 months. PET/CT scan, bone scan, and biopsy were recommended if clinically suspected of progression. The responses to therapy were assessed by an independent radiation oncologist and confirmed by a senior physician at 1–2 months after radiotherapy based on Response Evaluation Criteria in Solid Tumors version 1.1. Disease progression was documented according to clinical, radiographic, or pathological evidence, and the first failure patterns were recorded. Disease recurrence at primary tumor site or local–regional lymph node was considered as locoregional recurrence, and all other sites of recurrence or metastases were defined as distant metastasis. OS and progression-free survival (PFS) were defined as the time from the initiation of radiotherapy to death, and the first occurrence of disease progression or death, respectively. The last follow up ended at May 1st, 2020. Therapeutic toxicities were recorded according to the National Cancer Institute Common Toxicity Criteria (version 4.0).

### GTV risk group and the integrated GTV-TNM stratification system

The data on GTV were determined automatically from treatment planning system (TPS) using Zeus Cloud TPS V1.0 (Tongdiao, SuZhou, China). An X-tile analysis (Yale University, New Haven, CT, USA) provided the optimal outcome-based cut-off points to categorize patients into low GTV risk group (I), moderate GTV risk group (II) and high GTV risk group (III) in both the modeling and validation cohorts [15]. In order to optimize a novel integrated GTV-TNM stratification system, patients were classified into 9 subgroups: Group G1-IIIA (stage IIIA with GTV risk group I); Group G2-IIIA (stage IIIA with GTV risk group II); Group G3-IIIA (stage IIIA with GTV risk group III); Group G1-IIIB(stage IIIB with GTV risk group I); Group G2-IIIB (stage IIIB with GTV risk group II); Group G3-IIIB (stage IIIB with GTV risk group III); Group G1-IIIC (stage IIIC with GTV risk group I); Group G2-IIIC (stage IIIC with GTV risk group II); and Group G3-IIIC (stage IIIC with GTV risk group III). An ordered list of subgroups was constructed, relative to the best prognostic subgroup (Table 1). Several integrated stratification systems had been developed by combining similar subgroups [16, 17], and a final novel integrated GTV-TNM stratification system comprising three stratums was brought up, due to its statistical characteristics in the training cohort.

**Table 1:** Nine subgroups ordered by hazard ratio

Group	Sample size (training cohort)	Hazard ratio
G1-IIIB	32	1.00
G1-IIIA	21	1.71
G2-IIIA	37	2.17
G1-IIIC	12	2.41
G2-IIIB	58	2.46
G3-IIIA	7	3.60
G2-IIIC	24	3.67
G3-IIIC	12	5.17
G3-IIIB	24	6.35

### Statistical analysis

All eligible patients were randomized into a training cohort and a validation cohort with a ratio of 2:1. The distribution differences of categorical variables were examined with the Fisher’s exact test. Kaplan–Meier method was applied for survival analyses, which were compared by log-rank test (two-sided). Then those factors with a P value < 0.1 in the univariate analysis were incorporated into the Cox proportional hazards model to perform multivariate analysis for OS. A receiver operating characteristics (ROC) curve was produced for the integrated GTV-TNM stratification system, GTV risk group and TNM stage, and the area under ROC curve (AUC) was applied to assess the prognostic validity of these three different systems. Furthermore, bootstrap method was used to validate the predictions, which was done by randomly choosing 20% of the whole cohort for validation, and repeating 100 times to obtain a distribution of the prediction performance. For performance grading, F1 scores were calculated using Eq. 1–3 below:

1 $${\text{F}}_{1} = 2 \times \frac{{{\text{precision}} \times {\text{recall}}}}{{{\text{precision}} + {\text{recall}}}}$$

2 $${\text{precision}} = \frac{{{\text{TP}}}}{{{\text{TP}} + {\text{FP}}}}$$

3 $${\text{recall}} = \frac{{{\text{TP}}}}{{{\text{TP}} + {\text{FN}}}}$$

where TP is the true positive rate, FP is the false positive rate and FN is the false negative rate.

Statistical analyses were performed using SPSS 24.0 software (IBM, Chicago, IL, USA) and MATLAB® 2017 (MathWorks Inc., Massachusetts, USA), and the *P* value < 0.05 (two-sided) was considered as significant difference.

## Result

### Baseline characteristics

A total of 340 eligible patients were included in analysis. They were randomly assigned into a training group comprising 227 patients and a validation group with 113 patients. In the whole cohort, there were 64 females and 276 males with the median age of 58 (range from 28–81) years. There were 97 (28.5%) patients diagnosed with stage IIIA disease, 172 (50.6%) with stage IIIB and 71 (20.9%) with stage IIIC. For all patients, the median GTV volume was 101.0 (range, 9.1–664.3) cm^3^. In the training cohort, the optimal cutoff values of GTV in terms of OS were 71.2 cm^3^ and 177.2 cm^3^, which were determined by X-tile program. For the ease of clinical practice, we selected 70 cm^3^ and 180 cm^3^ as the uniform cutoff points in order to define patients into low, moderate and high GTV risk groups. GTV risk group I, II and III were defined as < 70 cm^3^, 70–180 cm^3^ and > 180 cm^3^, respectively. Docetaxel and platinum was the most commonly used regimen of concurrent chemotherapy (70.9%, 241/340). Most patients (254/340, 74.7%) received neoadjuvant chemotherapy before definitive CCRT, and 21.8% patients (74/340) underwent adjuvant chemotherapy. All clinic-pathologic characteristics were similarly distributed between the training and validation groups (Table 2).

**Table 2:** Patients characteristics

Characteristic	Training cohort	Validation cohort	*P* value
	n = 227 (%)	n = 113 (%)	
Sex			0.659
Male	186 (81.9)	90 (79.6)	
Female	41 (18.1)	23 (20.4)	
Age (y)			> 0.999
Median (range)	59 (28–79)	58 (31–81)	
ECOG score			0.126
0	29 (12.8)	16 (14.2)	
1	170 (74.9)	91 (80.5)	
2	28 (12.3)	6 (5.3)	
Weight loss ≥ 5 kg			0.153
Yes	23 (10.1)	6 (5.3)	
No	204 (89.9)	107 (94.7)	
Smoke index ≥ 400			0.298
Yes	129 (56.8)	57 (50.4)	
No	98 (43.2)	56 (49.6)	
Pathological types			0.627
Squamous cell carcinoma	130 (57.3)	65 (57.5)	
Adenocarcinoma	80 (35.3)	41 (36.3)	
Lymphoepithelioma-like carcinoma	10 (4.4)	5 (4.4)	
Adenosquamous carcinoma	1 (0.4)	1 (0.9)	
Adenoid cystic carcinoma	2 (0.9)	0 (0)	
Large cell neuroendocrine carcinoma	0 (0)	1 (0.9)	
Giant cell carcinoma	1 (0.4)	0 (0)	
Not otherwise specified(NOS)	3 (1.3)	0 (0)	
GTV (cm^3^)			> 0.999
Median(range)	100.6 (9.1–664.3)	101.3 (10.5–567.7)	
GTV risk group			0.993
I (< 70 cm^3^)	65 (28.6)	32 (28.3)	
II (70–180 cm^3^)	119 (52.4)	60 (53.1)	
III (> 180 cm^3^)	43 (19.0)	21 (18.6)	
TNM stage			0.978
IIIA	65 (28.6)	32 (28.3)	
IIIB	114 (50.2)	58 (51.3)	
IIIC	48 (21.2)	23 (20.4)	
Neoadjuvant chemotherapy			0.358
Yes	166 (73.1)	88 (77.9)	
No	61 (26.9)	25 (22.1)	
Adjuvant chemotherapy			0.127
Yes	55 (24.2)	19 (16.8)	
No	172 (75.8)	94 (83.2)	

ECOG, Eastern Cooperative Oncology Group; GTV, gross tumor volume

### Survival outcomes and tumor response

The median follow-up was 28.9 (range, 1.5–103.4) months in all patients and 46.6 (range, 6.7–103.4) months in event-free patients. Our analysis depicted a median estimated OS of 44.7 months in all patients, 45.5 months in the training group, and 38.1 months in the validation group. The 3-year and 5-year OS rate was 59.9% and 44.1% in the training set, compared with 52.9% and 38.0% in the validation set (*P* = 0.283), respectively. The median estimated PFS was 12.1, 13.0, and 10.4 months in the whole cohort, training cohort and validation cohort. The 1-year and 2-year PFS rate was 52.7% and 31.9% in the training group, versus 44.9% and 25.6% in the validation group (*P* = 0.181), respectively.

In the training cohort, 10 patients had complete remission (CR), 154 had partial remission (PR), 49 had stable disease (SD) and 14 had progressive disease (PD). In the validation cohort, 6 patients had CR, 82 had PR, 18 had SD and 7 had PD. The objective response rate (ORR) was 74.1%, 72.2% and 77.9% in the whole, training and validation cohort, respectively (Additional file 1).

### Risk factors for OS

Table 3 summarized the results of univariate analysis of OS based on data from the training set and validation set.

**Table 3:** Univariate analysis of risk factors for OS in the training cohort and validation cohort

Factor	Training cohort		Validation cohort	
	3-year OS (%)	*P* value	3-year OS (%)	*P* value
Sex		0.100		0.268
Male	57.9		50.1	
Female	69.6		65.1	
Age (y)		0.088		0.072
< 58	66.8		62.2	
≥ 58	54.4		44.9	
ECOG score		0.001		0.113
0	68.2		76.0	
1	63.1		49.1	
2	33.1		53.3	
Weight loss ≥ 5 kg		0.018		0.308
Yes	38.7		33.3	
No	62.2		54.1	
Smoke index ≥ 400		0.419		0.087
Yes	56.8		44.5	
No	64.2		62.0	
Pathological types		0.512		0.521
Squamous cell carcinoma	56.2		60.1	
Non-squamous cell carcinoma	65.5		59.4	
GTV risk group		< 0.001		< 0.001
I	79.2		85.5	
II	56.8		46.4	
III	37.4		18.5	
TNM stage		0.127		0.297
IIIA	58.7		59.6	
IIIB	65.3		49.5	
IIIC	49.7		53.2	
The integrated GTV-TNM stratification system		< 0.001		< 0.001
Stratum A	78.4		82.4	
Stratum B	63.7		56.8	
Stratum C	38.1		24.7	
Neoadjuvant chemotherapy		0.867		0.475
Yes	59.6		56.1	
No	60.9		41.3	
Adjuvant chemotherapy		0.382		0.713
Yes	55.7		52.6	
No	61.2		52.9	

ECOG, Eastern Cooperative Oncology Group; GTV, gross tumor volume

**Fig. 1:**
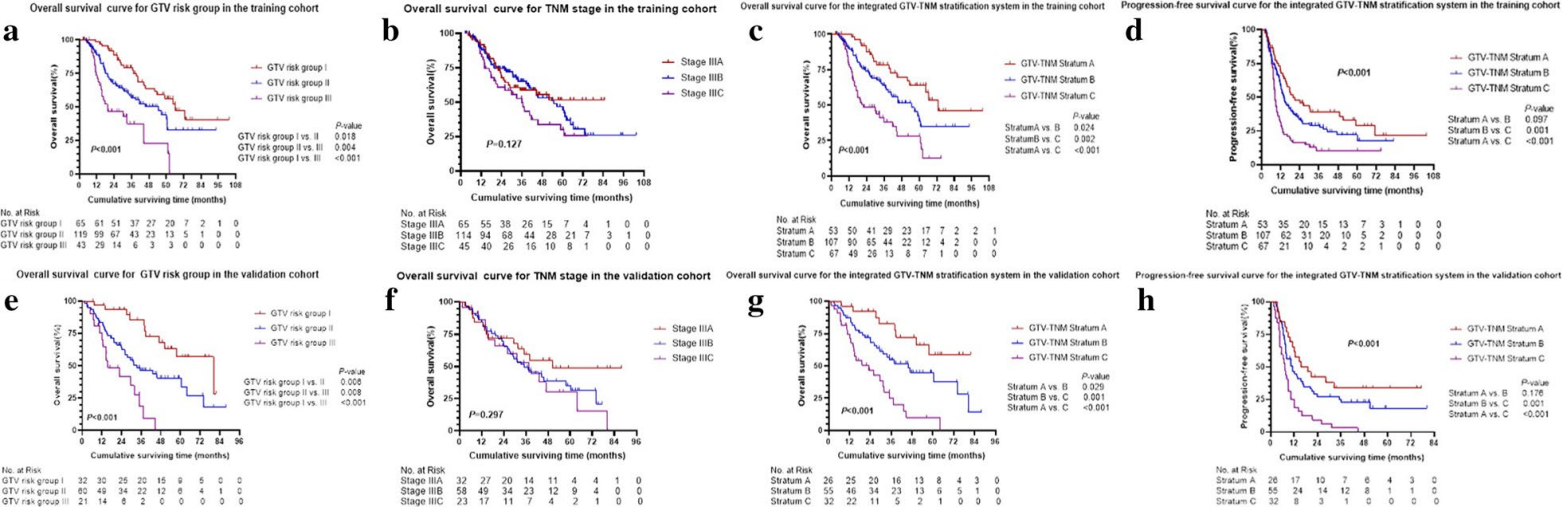
Survival outcomes. Overall survival (OS) curves for GTV risk group (**a**, **e**), TNM stage (**b**, **f**) and the integrated GTV-TNM stratification system (**c**, **g**), progression-free survival (PFS) curves for the integrated GTV-TNM stratification system (**d**, **h**), in the training cohort and validation cohort, respectively

In the training set, GTV risk group (3-year OS, group I vs II vs III, 79.2% vs 56.8% vs 37.4%, *P* < 0.001) (Fig. 1a), ECOG score (3-year OS, ECOG 0 vs 1 vs 2, 68.2% vs 63.1% vs 33.1%, *P* = 0.001), weight loss ≥ 5 kg (3-year OS, yes vs no, 38.7% vs 62.2%, *P* = 0.018) and age (3-year OS, < 58 years vs ≥ 58 years, 66.8% vs 54.4%, *P* = 0.088) were significantly associated with OS, while TNM stage failed to reach statistical significance (3-year OS, stage IIIA vs IIIB vs IIIC, 58.7% vs 65.3% vs 49.7%, *P* = 0.127) (Fig. 1b). In addition, neoadjuvant chemotherapy didn’t have tendency to improve OS (3-year OS, yes vs no, 59.6% vs 60.9%, *P* = 0.867). Then these four variables reached *P* < 0.1 in univariate analysis were further analyzed by using multivariate Cox proportional hazards model. Multivariate analysis demonstrated significant increased risk of death in GTV risk group II (hazard ratio (HR), 1.71; 95% confidence interval (CI), 1.06–2.78; *P* = 0.030) and group III (HR, 3.53; 95% CI 2.00–6.23; *P* < 0.001) compared with group I, and ECOG score (*P* = 0.032) was identified as another independent prognostic factor with HR of 1.61 (95% CI 1.04–2.49).

Similarly, in the validation group, univariate analysis revealed that patients with larger GTV (3-year OS, group I vs II vs III, 85.5% vs 46.4% vs 18.5%, *P* < 0.001) (Fig. 1e), older age (3-year OS, < 58 years vs ≥ 58 years, 62.2% vs 44.9%, *P* = 0.072) and smoke index ≥ 400 (3-year OS, yes vs no, 44.5% vs 62.0%, *P* = 0.087) had impaired OS. Again TNM stage (*P* = 0.297) (Fig. 1f) and neoadjuvant chemotherapy (*P* = 0.475) failed to predict OS. Only GTV risk group remained its statistical significance in multivariate analysis with HRs of 2.34 (95% CI 1.17–4.67; *P* = 0.016) and 6.27 (95% CI 2.78–14.16; *P* < 0.001) for group II and III relative to group I.

### Development and validation of a novel integrated GTV-TNM stratification system

In order to optimize a prognostic sub-staging system for LANSCLC undergoing CCRT by integrating GTV risk group with the current TNM stage, nine subgroups were categorized and ordered as mentioned above, which eventually brought up a novel integrated GTV-TNM stratification system comprising three stratums (Table 4): Stratum A (Group G1-IIIA–B); Stratum B (Group G2-IIIA–B and Group G1-IIIC); Stratum C (Group G2-IIIC and Group G3-IIIA–C).

**Table 4:**
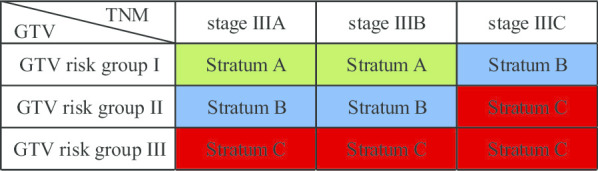
The integrated GTV-TNM stratification system

The integrated GTV-TNM stratification system (*P* < 0.001) significantly predicted OS in the training cohort, according to univariate analysis (3-year OS, Stratum A vs B, 78.4% vs 63.7%, *P* = 0.024; Stratum B vs C, 63.7% vs 38.1%, *P* = 0.002; Stratum A vs C, 78.4% vs 38.1%, *P* < 0.001) (Fig. 1c). Multivariate Cox regression analysis indicated increased risk of death in Stratum B (HR, 1.87; 95% CI 1.07–3.27; *P* = 0.028) and Stratum C (HR, 3.66; 95% CI 2.07–6.45; *P* < 0.001) compared with Stratum A, and ECOG score (*P* = 0.019) and weight loss ≥ 5 kg (*P* = 0.045) were independent prognostic factors of OS with HRs of 1.71 (95% CI 1.10–2.67) and 1.83 (95% CI 1.01–3.31), respectively (Additional file 2).

In the validation cohort, univariate Kaplan–Meier analysis demonstrated that the integrated GTV-TNM stratification system was statistically significantly associated with OS (3-year OS, Stratum A vs B, 82.4% vs 56.8%, *P* = 0.029; Stratum B vs C, 56.8% vs 24.7%, *P* = 0.001; Stratum A vs C, 82.4% vs 24.7%, *P* < 0.001) (Fig. 1g). Furthermore, the integrated GTV-TNM stratification system was recognized as the only independent prognostic factor for OS in multivariate analysis with HRs of 2.25 (95% CI 1.02–4.97; *P* = 0.045) and 5.62 (95% CI 2.46–12.80; *P* < 0.001) for Stratum B and C relative to Stratum A.

### The prognostic validity of the integrated GTV-TNM stratification system

**Fig. 2:**
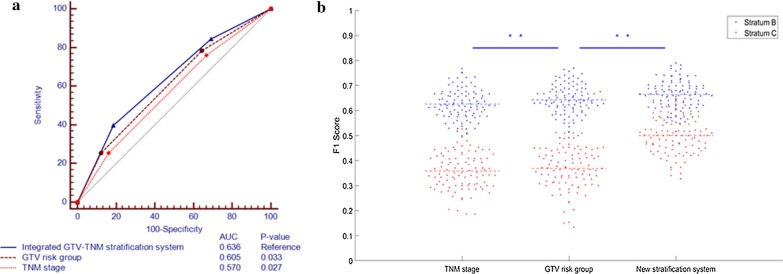
The prognostic validity of the integrated GTV-TNM stratification system, GTV risk group and TNM stage. **a** The ROC curve comparing these three stratification systems. **b** F1 score distributions acquired with bootstrap method. Colors indicate validations using Stratum B and Stratum C as predictors for survival outcome. Stars indicate significance between two groups

We used ROC curve to evaluate the prognostic validity of the integrated GTV-TNM stratification system, comparing with TNM stage and GTV risk group. In all patients, the AUC for OS was 0.636 (95%CI, 0.577–0.695) for the integrated GTV-TNM stratification system, versus 0.570 (95%CI, 0.509–0.631; *P* = 0.027) for TNM stage and 0.605 (95%CI, 0.545–0.665; *P* = 0.033) for GTV risk group (Fig. 2a). Bootstrap analysis demonstrated a significant increasing F1-scores in the integrated GTV-TNM stratification system (0.655 ± 0.052), compared to GTV risk group (0.638 ± 0.054, *P* = 0.013) and TNM stage (0.615 ± 0.056, *P* < 0.001), respectively (Fig. 2b).

### First failure patterns and the prognosis of PFS

With a median follow-up of 28.9 months, a total of 238 patients exhibited failure, but 9 patients had no record of failure pattern. The most common type of first failure pattern is distant metastasis (58.0%, 192/331), while the rate of locoregional recurrence was 39.9% (132/331). Meanwhile, 25.1% (83/331) patients had synchronous failure experiencing distant metastasis and locoregional recurrence at the same time (Table 5). The integrated GTV-TNM stratification system had significant association with failure (*P* = 0.018) and distant metastasis (*P* = 0.023), and Stratum C had a higher risk to experience failure and distant metastasis.

**Table 5:** The distribution of first failure patterns among patients with the integrated GTV-TNM stratification system in the whole cohort

The integrated GTV-TNM stratification system	First failure patterns							
	Locoregional recurrence (%)	*P* value	Distant metastasis (%)	*P* value	Synchronous failure (%)	*P* value	Total (%)	*P* value
Stratum A (n = 79)	28 (35.4)	0.393	40 (50.6)	0.023	18 (22.9)	0.222	50 (63.3)	0.018
Stratum B (n = 157)	61 (38.9)		86 (54.8)		35 (22.3)		110 (70.1)	
Stratum C (n = 95)	43 (45.3)		66 (69.5)		30 (31.6)		78 (82.1)	
Total (n = 331)^a^	132 (39.9)		192 (58.0)		83 (25.1)		238 (71.9)	

^a^9 patients had no record of failure pattern, but their deaths were documented, thus a total of 331 patients were enrolled in this analysis of first failure patterns

GTV, gross tumor volume

In the prediction of PFS, univariate analysis showed that patients with higher stratum in the integrated GTV-TNM stratification system tended to have impaired PFS in both the training group (1-year PFS, Stratum A vs B vs C, 67.5% vs 58.8% vs 31.3%, *P* < 0.001) (Fig. 1d) and validation group (1-year PFS, Stratum A vs B vs C, 65.4% vs 46.6% vs 25.0%, *P* < 0.001) (Fig. 1h). Even though the prognosis failed to reach statistical significance when Stratum A vs Stratum B (*P* = 0.097 and *P* = 0.176, respectively), the integrated GTV-TNM stratification system could be a potential strong prognostic factor for PFS.

### Therapeutic toxicities

The documented therapeutic toxicities were mostly grade 1 or 2. Forty-three cases (12.6%) of grade 3–5 acute pneumonitis were reported in our cohorts, including two patients with grade 5 pneumonitis. There were 16.5% of patients (56/340) developed grade 3–4 radiation esophagitis, and 17.9% patients (61/340) had grade 3–4 myeloctoxicity. The integrated GTV-TNM stratification system significantly correlated with Grade ≥ 3 pneumonitis (*P* = 0.026), and Stratum C had a higher risk to develop Grade ≥ 3 pneumonitis (Table 6).

**Table 6:** Grade ≥ 3 therapeutic toxicities among patients with the integrated GTV-TNM stratification system in the whole cohort

The integrated GTV-TNM stratification system	Toxicities					
	Grade ≥ 3 pneumonitis (%)	*P* value	Grade ≥ 3 esophagitis (%)	*P* value	Grade ≥ 3 myeloctoxicity (%)	*P* value
Stratum A (n = 79)	7 (8.9)	0.026	10 (12.7)	0.395	15 (19.0)	0.857
Stratum B (n = 162)	16 (9.9)		26 (16.0)		30 (18.5)	
Stratum C (n = 99)	20 (20.2)		20 (20.2)		16 (16.2)	
Total (n = 340)	43 (12.6)		56 (16.5)		61 (17.9)	

## Discussion

Our results demonstrate that the prognostic stratification system integrated GTV with TNM staging has provided greater predictive value for survival outcomes in unresectable LANSCLC patients receiving definitive CCRT. To our best knowledge, it is the first study to propose an integrated GTV-TNM stratification system and depict its prognostic value, with the largest sample size to date.

The current study used the optimal outcome-based GTV cutoff points (70 and 180 cm^3^) of the modeling cohort to determine each patient’s GTV risk group. Our results suggested that GTV was a strong prognostic factor in unresectable LANSCLC, which were consistent with previous studies [18–20]. Then we established a novel integrated GTV-TNM stratification system, which was examined in the modeling and validation cohorts using Kaplan–Meier method and Cox regression. The univariate analysis indicated that the integrated GTV-TNM stratification system significantly predicted OS in the training cohort (3-year OS, Stratum A vs B, 78.4% vs 63.7%, *P* = 0.024; Stratum B vs C, 63.7% vs 38.1%, *P* = 0.002; Stratum A vs C, 78.4% vs 38.1%, *P* < 0.001). Multivariate Cox regression analysis showed an increased risk of death in Stratum B (HR, 1.87; 95% CI 1.07–3.27; *P* = 0.028) and Stratum C (HR, 3.66; 95% CI 2.07–6.45; *P* < 0.001) compared with Stratum A. In the prediction of PFS, patients with higher stratum in the integrated GTV-TNM stratification system tended to have impaired PFS (*P* < 0.001). Such statistical significance could achieve in the validation group equally. The results were robust in these two randomized sets, suggesting that the integrated GTV-TNM stratification system may be able to generalize to the general population.

Several clinical and pathological characteristics have been considered as prognostic factors for survival outcomes. Recently multiple published studies have demonstrated that pretreatment tumor burden measured by PET/CT scan was highly correlated with treatment outcomes in LANSCLC patients receiving definitive CCRT [21–23]. Bradley et al. [18] concluded that GTV determined by three-dimensional conformal radiotherapy (3DCRT) planning had great prognostic value for long-term survival and local control. In the secondary analysis of the Radiation Therapy Oncology Group (RTOG) 93-11, Werner-Wasik et al. [19] reported that an increasing GTV was strongly associated with poor OS and PFS. In addition, Basaki et al. [20] suggested that GTV included primary tumor volume and positive lymph nodes, could provide better prognostic value for survival outcomes than TNM stage alone, which was consistent with several published reports [24, 25] and the present study. Moreover, the relationship between tumor volume and tumor control probability for NSCLC, breast tumor, head and neck cancer, malignant melanoma, and cervical carcinoma had been extensively investigated by clinical data as well as radiobiologic models [26–29]. However, the majority of those studies were conducted retrospectively, and several published data didn’t support the prognostic value of tumor volume [30–32]. Ball et al. [33] launched a multicenter prospective observational study to investigate the prognostic value of primary tumor volume in stage I-III NSCLC treated by definitive radiotherapy. They concluded that primary tumor volume failed to provide additional prognostic information after adjusting for the effects of the T and N stages. This may indicate that making prognosis with tumor volume alone is not adequate. Our results suggested GTV was an independent prognostic factor. The AUC the prediction of for OS was 0.636 for the integrated GTV-TNM stratification system, versus 0.570 for TNM stage (*P* = 0.027) and 0.605 for GTV risk group (*P* = 0.033), which implied tumor volume alone was not sufficient for prediction of LANSCLC patients after CCRT.

Therefore, TNM staging for LANSCLC remains useful for classifying the extent of spread of cancer, while GTV appears to be a crucial factor for CCRT. Although TNM staging is more commonly regarded as a classification system of anatomical extent of tumor, it provides great details of the spread route of lung cancer. In TNM staging, the “T” classification comprises the size, location and extent of the primary tumor, while the “N” classification represents lymph node involvement, indicating whether or not the lymph nodes are infiltrated and the involved lymph node region and station. It could perfectly illustrate the relationship between primary tumor and involved lymph nodes in LANSCLC. It would be more practical to integrate the GTV information into the current TNM classification system than GTV risk group or TNM staging alone in LANSCLC patients treated with CCRT. The integration would effectively guide the clinical management and develop better treatment approaches for this subgroup of patients. Other than that, intensity-modulated radiotherapy (IMRT), especially SMART technique, has become the leading radiotherapy technique of lung cancer substituting for 3DCRT. SMART technique was used to deliver different prescription doses to GTV and CTV simultaneously, which afforded dose escalation to GTV and sparing of surrounding tissues. Furthermore, treatment planning in radiotherapy is currently conducted on specialized computerized planning system, which could enable exact location, definition and quantification of tumor volume.

The ROC curve results suggested that the prognostic validity could be increased by integrating the current TNM staging system with GTV. Analysis using the ROC curve is typically more suitable for models using continuous predictor for survival outcome, whereas in our study, the predictor used is discrete (either Stratum B or Stratum C). Thus we employ a more suitable index, i.e. F1-score, which makes full use of precision and recall (or sensitivity) to evaluate the model performance, and is routinely used in the field of computer science. In our study, F1-score was significantly higher in the integrated GTV-TNM stratification system and indicated a superior prognostic value comparable to TNM stage and GTV risk group alone in LANSCLC patients treated with definitive CCRT.

It was well recognized that consolidation chemotherapy failed to yield significant survival benefit for unresectable LANSCLC [34–38]. However, based on the series results of the phase III PACIFIC study and other phase II trials [39–41], consolidation immunotherapy following CCRT significantly improve PFS and OS with acceptable toxicities in these patients, establishing a new standard of care. In PACIFIC trial, the PFS curve of durvalumab group plateaued at a proportion surviving of 40%, indicating that certain subpopulation could achieve a potential cure. In addition, compared with placebo, a higher incidence of pneumonitis or radiation pneumonitis of any grade occurred in patients receiving durvalumab (33.9% vs. 24.8%), but the rate of grade 3 or 4 was similar (3.4% vs. 2.6%). To be noticed, Asian patients seemed to have a higher incidence of any grade pneumonitis (73.6%) and severe pneumonitis (5.6%). Identifying the high-risk subgroup early is essential for the decision of consolidation immunotherapy, based on the concept of individualized treatment. Our results indicated that patients with the integrated GTV-TNM stratification system Stratum C had a higher risk to develop severe pneumonitis. Therefore, because of the lung injury following large-volume irradiation, consolidation immunotherapy might not be safely administered in Stratum C patients, which deserves further investigation.

Honestly, there were several limitations existed in our retrospective study. Firstly, it was a study comprised patients from 2 clinical trials and daily clinical practice, thus the concurrent chemotherapy regimens were determined by specific protocols and individual patient management. Secondly, the cut-off values for GTV risk group and the integrated GTV-TNM stratum were identified from a single center. Despite the distribution of TNM stages in our analysis seems to be consistent to the global sample [16], larger external databases are warranted to validate the optimal cut-off values for the general population of LANSCLC patients.

## Conclusions

We proposed a novel integrated GTV-TNM stratification system to supplement unresectable LANSCLC sub-staging due to its prognostic value independent of TNM stage and other clinical characteristics, suggesting that it could be considered in individual treatment decision-making process.

## Supplementary information

**Additional file 1**. Tumor response.

**Additional file 2**. Multivariate analysis of the prognosis of the integrated GTV-TNM stratification system in the training cohort and validation cohort.

## Data Availability

The datasets used and/or analysed during the current study are available from the corresponding author on reasonable request.

## Abbreviations

GTV: Gross tumor volume
LANSCLC: Locally advanced non-small cell lung cancer
CCRT: Concurrent chemoradiotherapy
AUC: Area under the receiver operating characteristic curve
NSCLC: Non-small cell lung cancer
OS: Overall survival
SMART: Simultaneous modulated accelerated radiation therapy
ECOG: Eastern Cooperative Oncology Group
CT: Computed tomography
MRI: Magnetic resonance imaging
PET/CT: Positron emission tomography/computed tomography
4DCT: 4-Dimensional computed tomography
MIP: Maximum intensity projection
CTV: Clinical target volume
PTV: Planning target volume
OAR: Organ at risk
EBUS-TBNA: Endobronchial ultrasound-transbronchial needle aspiration
CBCT: Cone beam computed tomography
TKI: Tyrosine kinase inhibitor
PFS: Progression-free survival
TPS: Treatment planning system
ROC: Receiver operating characteristics
CR: Complete remission
PR: Partial remission
SD: Stable disease
PD: Progressive disease
ORR: Objective response rate
HR: Hazard ratio
CI: Confidence interval
RTOG: Radiation Therapy Oncology Group
IMRT: Intensity-modulated radiotherapy
