# Supplementary material for: Developing and validating an integrated gross tumor volume (GTV)-TNM stratification system for supplementing unresectable locally advanced non-small cell lung cancer treated with concurrent chemoradiotherapy
Source: Radiat Oncol. 2020 Nov 10;15:260. doi: 10.1186/s13014-020-01704-2 (PMC7653712; doi:10.1186/s13014-020-01704-2)
Supplement: Supplementary file 1 — Additional file 1. Tumor response. [file 13014_2020_1704_MOESM1_ESM.docx]

**Additional File 1** Tumor response

| response | The training cohort (n, %) | | | | *P*-value | The validation cohort (n, %) | | | | *P*-value |
| --- | --- | --- | --- | --- | --- | --- | --- | --- | --- | --- |
|  | Stratum A  (n=53) | Stratum B  (n=107) | Stratum C  (n=67) | Total  (n=227) | <0.001 | Stratum A  (n=26) | Stratum B  (n=55) | Stratum C  (n=32) | Total  (n=113) | 0.130 |
| PD | 2(3.8) | 6(5.6) | 6(9.0) | 14(6.2) |  | 2(7.7) | 2(3.6) | 3(9.4) | 7(6.2) |  |
| SD | 5(9.4) | 29(27.1) | 15(22.4) | 49(21.6) |  | 3(11.5) | 6(10.9) | 9(28.1) | 18(15.9) |  |
| PR | 38(71.7) | 70(65.4) | 46(68.7) | 154(67.8) |  | 18(69.2) | 44(80.0) | 20(62.5) | 82(72.6) |  |
| CR | 8(15.1) | 2(1.9) | 0(0) | 10(4.4) |  | 3(11.5) | 3(5.5) | 0(0) | 6(5.3) |  |
| ORR | 46(86.8) | 72(67.3) | 46(68.7) | 164(72.2) |  | 21(80.8) | 47(85.5) | 20(62.5) | 88(77.9) |  |
