# Supplementary material for: Developing and validating an integrated gross tumor volume (GTV)-TNM stratification system for supplementing unresectable locally advanced non-small cell lung cancer treated with concurrent chemoradiotherapy
Source: Radiat Oncol. 2020 Nov 10;15:260. doi: 10.1186/s13014-020-01704-2 (PMC7653712; doi:10.1186/s13014-020-01704-2)
Supplement: Supplementary file 2 — Additional file 2. Multivariate analysis of the prognosis of the integrated GTV-TNM stratification system in the training cohort and validation cohort. [file 13014_2020_1704_MOESM2_ESM.docx]

**Additional File 2** Multivariate analysis of the prognosis of the integrated GTV-TNM stratification system in the training cohort and validation cohort

|  | Factor | HR | 95% CI for HR | *P-*value |
| --- | --- | --- | --- | --- |
| Training cohort | ECOG score | 1.71 | 1.10-2.67 | 0.019 |
|  | Weight loss ≥5Kg | 1.83 | 1.01-3.31 | 0.045 |
|  | The integrated GTV-TNM stratification system |  |  | <0.001 |
|  | Stratum B vs Stratum A | 1.87 | 1.07-3.27 | 0.028 |
|  | Stratum C vs Stratum A | 3.66 | 2.07-6.45 | <0.001 |
| Validation cohort | The integrated GTV-TNM stratification system |  |  | <0.001 |
|  | Stratum B vs Stratum A | 2.25 | 1.02-4.97 | 0.045 |
|  | Stratum C vs Stratum A | 5.62 | 2.46-12.80 | <0.001 |

ECOG=Eastern Cooperative Oncology Group, GTV=gross tumor volume, HR=hazard ratio.
